# Mutational Robustness of Gene Regulatory Networks

**DOI:** 10.1371/journal.pone.0030591

**Published:** 2012-01-25

**Authors:** Aalt D. J. van Dijk, Simon van Mourik, Roeland C. H. J. van Ham

**Affiliations:** 1 Applied Bioinformatics, PRI, Wageningen UR, Wageningen, The Netherlands; 2 Biometris, Plant Sciences Group, Wageningen UR, Wageningen, The Netherlands; 3 Netherlands Consortium for Systems Biology (NCSB), Amsterdam, The Netherlands; Lehigh University, United States of America

## Abstract

Mutational robustness of gene regulatory networks refers to their ability to generate constant biological output upon mutations that change network structure. Such networks contain regulatory interactions (transcription factor – target gene interactions) but often also protein-protein interactions between transcription factors. Using computational modeling, we study factors that influence robustness and we infer several network properties governing it. These include the type of mutation, i.e. whether a regulatory interaction or a protein-protein interaction is mutated, and in the case of mutation of a regulatory interaction, the sign of the interaction (activating vs. repressive). In addition, we analyze the effect of combinations of mutations and we compare networks containing monomeric with those containing dimeric transcription factors. Our results are consistent with available data on biological networks, for example based on evolutionary conservation of network features. As a novel and remarkable property, we predict that networks are more robust against mutations in monomer than in dimer transcription factors, a prediction for which analysis of conservation of DNA binding residues in monomeric vs. dimeric transcription factors provides indirect evidence.

## Introduction

Transcription factors (TFs) regulate gene expression by binding to DNA adjacent to target genes. In addition, TF proteins often physically interact with each other and form protein dimers, which in combination with regulatory interactions gives rise to an intricate network of interactions. Aspects of TF-TF protein-protein interactions and how these change during evolution have been studied [1], [2], [3], and dimerization is known to be important for various TF families [4]. Known or presumed biological roles of TF dimerization include potential effects at the level of DNA recognition, such as facilitated proximity and enhancement of DNA-binding specificity. Roles at the level of the network output include that dimerization might function to dampen noise due to fluctuations in monomer concentrations [5], or might be important in attaining multistability in certain types of networks [6]. Dimerization could also serve as a means to generate ultrasensitive responses via molecular titration, which occurs when an active subunit is sequestered into an inactive heterodimer complex by a titrating molecule [7]. Depending on interaction strength, dimerization also influences the kinetics of the TF-DNA search process [8].

Perturbations of gene regulatory networks, either through input concentrations, parameters of the interactions, or the network topology, can result in changes in network output, i.e. the resulting expression pattern. Robustness refers to the stability of this output in response to perturbations, and although robustness against perturbations of concentrations or against parameter changes has obtained a lot of attention [9], [10], [11], [12], [13], [14], [15], [16], [17], [18], the effect of evolutionary tinkering with network structure via changes in the sequences of the underlying components has received less attention [18], [19], [20], [21], [22]. This is true in general, but in particular for networks which contain dimerizing TFs. Given the biological importance of TF dimerization as explained above, better understanding of robustness of such networks would be valuable.

Computational studies of the effects of network rewiring have been performed mainly using discrete approaches such as Boolean Networks (BNs) or related models. Robustness and evolvability of a BN under the process of gene duplication followed by divergence was studied, and it was found that networks operating close to the ‘critical regime’, which depends on the network connectivity, exhibit the maximum robustness and evolvability simultaneously [23]. Several studies focused on the relation of robustness to mutations with robustness to noise [19], [20]. These two were shown to be highly correlated [24]. In addition, nearly all networks can evolve toward greater robustness through gradual changes in topology. It was also shown that two networks with exactly the same phenotype may produce very different innovations, depending on their topology [22].

Importantly, various experimental analyses indicate that gene regulatory networks can be highly robust against network rewiring [25], [26], [27]. However, network properties influencing it are not clearly understood. Here, we analyze the relation between network properties and mutational robustness using a computational approach, in combination with an integrative analysis of various experimental datasets.

## Results

**Figure 1:**
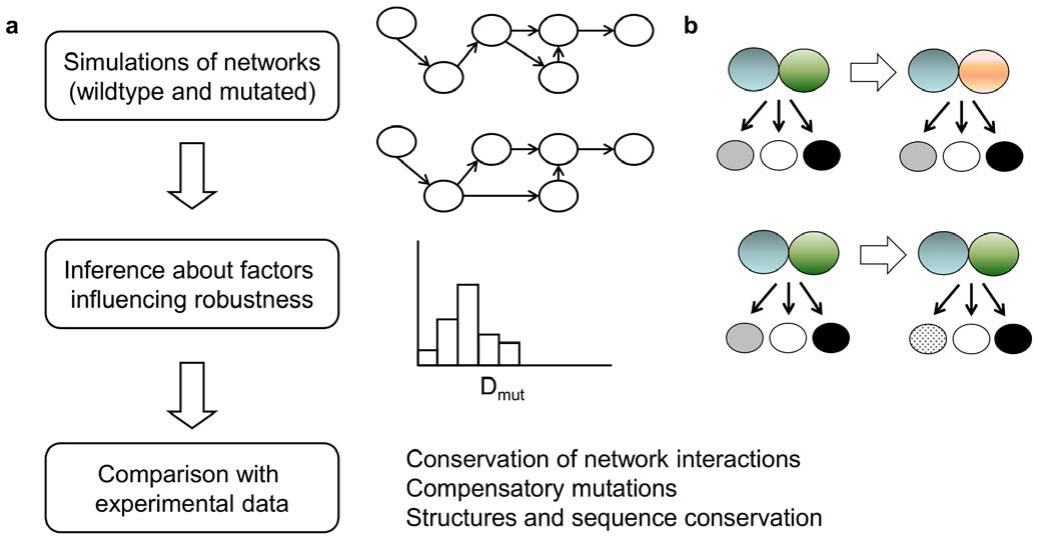
Overview of our study of evolutionary robustness. (**a**) Models for gene regulatory networks containing transcription factor – transcription factor (protein-protein) interactions and transcription factor – target gene interactions were simulated. Mutations changing the network topology were applied, and the resulting change in expression patterns was described using a metric D_mut_. By comparing networks with various properties, network features were found that influence network robustness. Finally, the observed trends were compared with available data about biological networks. (**b**) Two types of mutations were applied, either targeting a protein-protein interaction (top panel), or targeting a regulatory interaction (bottom panel). In the case of a mutation changing a protein-protein interaction, a dimer is changed into another dimer; all its regulatory interactions remain. In the case of a mutation changing a regulatory interaction, for one specific regulator (either a dimer as shown in the figure, or a monomer in case of monomeric networks), one regulatory interaction is changed; all other regulatory interactions remain, as do all protein-protein interactions.

We investigated robustness against mutations in large ensembles of simulated network models (Figure 1). In these networks, two types of interactions were present: TF-target gene (regulatory) interactions, and TF-TF protein-protein interactions. In contrast with previous studies of robustness and evolvability that used discrete models, we used ordinary differential equations to simulate networks. Each ensemble was constructed using specified topology parameters and within ensembles, TF-TF and TF-target gene interaction parameters were sampled. We applied two types of mutations: protein-protein interaction mutations and mutations of regulatory interactions. These mutations were designed such that, for a protein interaction mutation, all regulatory interactions were retained, and for a regulatory interaction mutation, all protein interactions were retained. The difference between the resulting expression pattern (network output) of mutated vs. wildtype networks was represented by the metric D_mut_; thresholds for “small” and “large” differences, D_small_ and D_large_, were derived from experimental data and are described in the first part of the Results section. After that, we describe the observations from our simulations, followed by comparison with experimental data.

### Expression data analysis and D_mut_ cutoffs

In our analysis of robustness of gene regulatory networks against network rewiring, we want to characterize whether differences in expression between two network variants are ‘large’ or ‘small’. We use a metric D_mut_ to compute the difference between two such expression patterns. An important methodological question is how to determine a suitable cutoff for D_mut_ such that the difference between the expression patterns is “small” or “large”. The need for a threshold value to define whether a mutation has a large influence on network output arises because we use a continuous instead of the previously used discrete approaches to study robustness. In the latter, one typically finds the stable states of wildtype and mutated networks and compares those to see whether they are exactly identical or not. This is however not directly applicable in our case.

**Figure 2:**
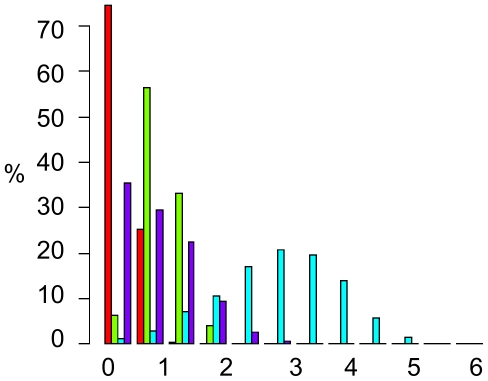
Histogram of D_mut_ for experimental datasets. D_mut_ quantifies expression differences between orthologs (interspecies sets) or the same gene in various conditions (intraspecies sets). Interspecies analysis was performed using wine yeast strains (red) and *Drosophila* species (green), and intraspecies analysis for *Arabidopsis thaliana* (blue) and human (purple). The observed 1th and 99th percentile were used to obtain cutoffs for D_mut_ in order to describe ‘small’ and ‘large’ changes (D_small_ and D_large_).

To answer the question how to define threshold values for D_mut_, we computed D_mut_ values obtained by analyzing a number of different gene expression datasets. We performed two inter-species comparisons, where we compared expression data for four different wine yeast strains [28] and for two *Drosophila* species [29], as well as two intra-species comparisons (comparing different cell types) within *Arabidopsis thaliana* [30] and within human [31] (Figure 2). To define a cutoff for ‘large effect’, we reasoned that at least some of the differences in expression of network components between species must be responsible for differences between those species. We obtained the D_mut_ observed in the cross-species set such that 99% of the cases have a lower D_mut_. For the wine yeast strains, 99% of the cases have a D_mut_ below 0.92, and for the two *Drosophila* species (which are at a larger evolutionary distance) this is 1.7. We take a value between those two values as a cutoff for “large effect” and set this cutoff at D_large_ = 1.0.

To obtain a lower limit for D_mut_ below which we could say that the effect is likely to be small, we reasoned that at least some networks would be expected not to change between various conditions or tissues within one species. Hence, we analyzed the two datasets of expression between various conditions/tissues within one species. Here, in the human dataset, 99% of the cases had D_mut_ above 0.1, and for the Arabidopsis dataset 99% had D_mut_ above 0.5. Note that the Arabidopsis dataset is more targeted (a small number of factors known to be involved in the process of flowering was analyzed, for which the different tissues selected from the dataset are the relevant ones). We take again a value in between and set this cutoff at D_small_ = 0.2. For the wineyeast set a cutoff based on similar reasoning would be 0.11, and for *Drosophila* 0.33, which are compatible with this value.

We are aware of the fact that our analysis here does not give a definite answer on how to define whether a gene regulatory network mutation has a “large” effect. However, to a certain extent we circumvent the associated problems by comparing different types of changes with each other, which means that we analyze relative D_mut_ values instead of their absolute values. Importantly, the main conclusions from our analysis were robust against the exact value of the defined cutoffs (Text S1, Table S1).

### Simulation results

To simulate gene regulatory networks, systems of ordinary differential equations (ODEs) were used, which describe TF-TF dimerization and TF-target gene regulation (via Hill functions). To investigate the stability upon network rewiring, for each network, one ‘wildtype’ simulation was done, and two simulations of mutated versions. One mutated network was obtained with a protein-protein interaction change, where a dimer A–B is changed to dimer A–C (that did not occur yet in the protein-protein interaction network). All other dimers are preserved, and this new dimer preserves the regulatory interactions of the original dimer. A second mutated version of the network was obtained with a regulatory change where for one specific dimer, one of the genes that it regulates is changed to another gene (which it did not yet regulate). In this case, none of the protein-protein interactions is changed. For monomeric networks, only a mutation in a regulatory interaction was applied. The way in which these mutations are constructed ensures that the number of interactions does not change, which allows a more fair comparison between wildtype and mutated system than in case this number would change. The resulting stable state concentrations after simulating the mutated networks were compared with the wildtype concentrations using D_mut_. If networks with certain properties obtain in most cases a low D_mut_ upon certain types of mutations, then these networks have a high robustness against that particular type of mutations, and vice versa.

Theoretical results for e.g. Boolean Networks or related models are often reported with densely connected networks (many regulatory interactions per gene) but this means that adding spurious interactions is rewarded in the sense that mutating such interactions will not lead to phenotypic changes, resulting in an artificially increased robustness. Because of that, we chose values for fractions of regulatory interactions that are close to what was found in various experimental networks [32]. We also distinguish ensembles of networks based on their connectivity, making a proper comparison between networks with similar connection density. The fraction of dimers (F_dim_) in our networks equaled 0.0 (monomeric networks), 0.3, or 0.6, and the fraction of regulatory interactions (F_regint_) equaled 2.0 or 4.0. Here F_dim_ was defined as the fraction of dimers out of all putatively possible dimers, and F_regint_ as the average number of TFs regulating the expression of a given target. We also separately analyzed networks with different fractions of activating interactions (0.25, 0.5, and 0.75) but because these resulted in overall similar observations (Supporting Information Figure S1), the analysis below focusses on networks with fraction activating interactions 0.5.

**Figure 3:**
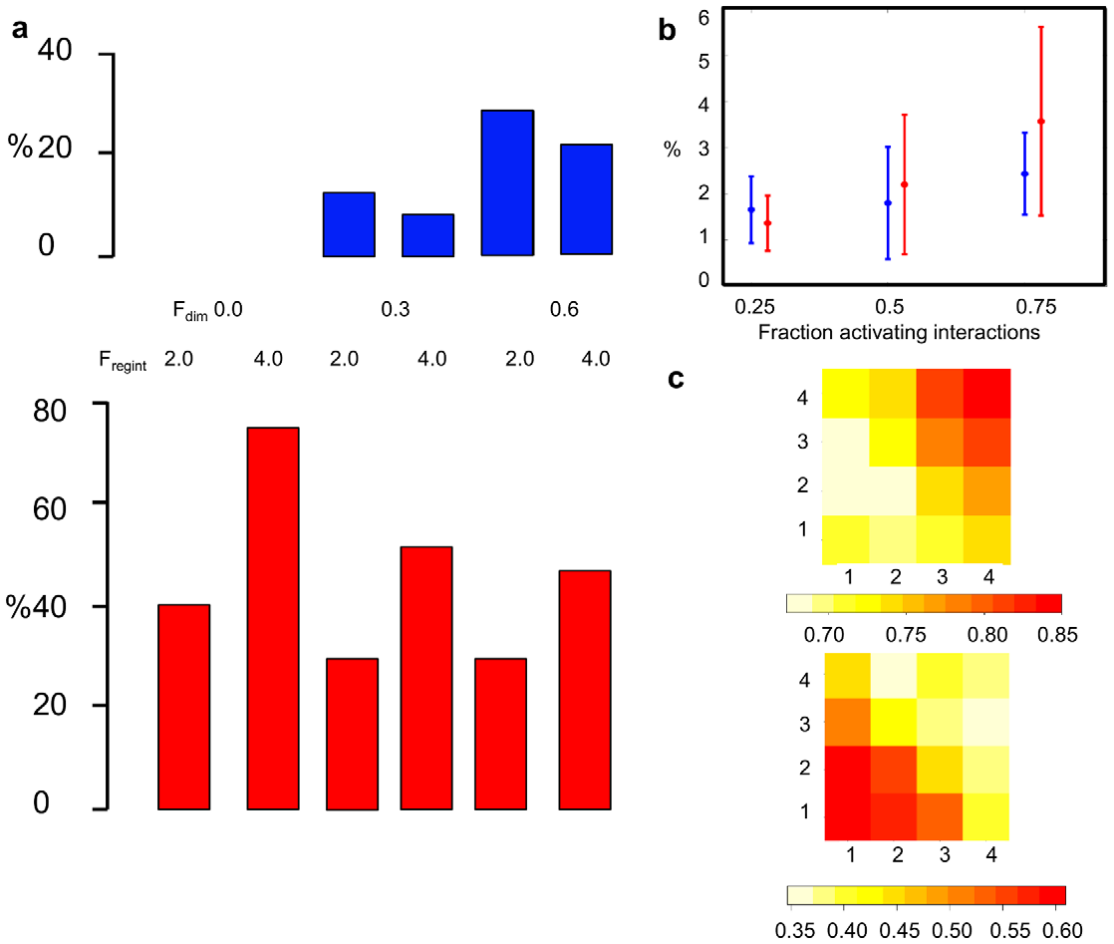
Variation of gene regulatory network robustness. (**a**) Percentage of networks whose output does not change upon mutation (D_mut_<D_small_). Fraction of dimers (F_dim_) equals 0.0, 0.3 or 0.6, and fraction regulatory interactions (F_regint_) equals 2.0 or 4.0. *Upper panel*, protein interaction mutations. *Lower panel*, regulatory mutations. (**b**) Percentage of compensatory mutations after a first large effect (D_mut_>D_large_) protein interaction mutation. From left to right, the fraction activating interactions equals 0.25, 0.5 or 0.75. Blue symbols indicate average percentage (and standard deviation) for secondary protein interaction mutations, red for secondary regulatory mutations. (**c**) Dependence of robustness on number of autoregulatory interactions. Average D_mut_ is shown (legend at the bottom) depending on the number of activating (x-axis) and repressing (y-axis) autoregulatory interactions, for protein interaction mutations (*upper panel*) and regulatory mutations (*lower panel*). Note that the higher the average D_mut_, the lower the robustness.

We made five key observations on network robustness (Table 1). First, protein interaction mutations are more likely to have a large effect than mutations in regulatory interactions, especially in case of a low fraction of dimers (Figure 3A). Using a randomization procedure, this difference in robustness was statistically significant (p<0.001) for three of the four cases shown; only for the case with F_dim_ 0.6, F_regint_ 4.0 it was not significant (p∼0.1). Second, repressive interactions are associated with a higher robustness against protein-protein interaction mutations as well as regulatory interaction mutations, compared to activating interactions. This was assessed by counting the number of repressive vs. activating interactions that the regulator affected by the mutation was involved in. In particular, when the regulator was only involved in repressive interactions, averaged over the different ensembles, the average (standard deviation) of D_mut_ was only 0.39+/−0.47, whereas when the regulator was only involved in activating interactions, it was 0.62+/−0.46. Similar but more pronounced differences were obtained when analyzing this effect for each of the different values of F_dim_ and F_regint_ separately (Text S1, Table S2).

**Table 1:** Predicted effects of various network characteristics on robustness^a^.

Feature	Effect on Robustness
Regulatory vs. protein interaction mutation	More robustness against regulatory mutations
Mutation of activating vs. repressive interaction	More robustness against mutation of repressive interaction
Combinations of mutations	Compensatory effects
Monomer vs. dimer networks	Monomer networks are more robust
Number of auto-regulatory interactions	Opposing effects on robustness against regulatory or protein interaction mutations

a Simulations were performed with models of gene regulatory networks, both for wildtype and mutated versions of the network. This enabled to find characteristics of networks with low robustness (large changes in expression patterns upon mutations) vs. those with high robustness (small changes in expression patterns upon mutations).

Third, to explore the interdependency of mutations, we tested for possible compensatory effects, i.e. two subsequent mutations that in combination result in a small net change to the original expression pattern although the first mutation has a large effect. Because protein interaction mutations had the largest effect, our starting point was protein interaction mutations with high impact (D_mut_>D_large_). When the follow-up mutation is again a protein interaction mutation, the overall robustness for the combination of the two mutations does not depend much on the fraction of activating interactions. When the next mutation is a regulatory mutation, however, the percentage of compensatory mutations increases with increasing amount of activating interactions; at the highest fraction of activating interactions, a given regulatory mutation has a ∼3.5% chance of ‘rescuing’ a previous large-effect protein interaction mutation (Figure 3B). The exact value of this percentage depends on the value of the thresholds for D_mut_ defined above (for example, with a stricter threshold it is ∼1%) but the trend observed in Figure 3B stays the same. The fact that we find computational evidence for such compensatory mutations is novel, and as discussed below, there are various experimental indications that compensatory mutations indeed exist. Fourth, networks without dimers are much more robust against changes in regulatory interactions (Figure 3A). Using a randomization procedure, this difference in robustness was statistically significant (p<0.001) for each of the comparisons between monomeric and dimeric networks with the same fraction of regulatory interactions. Finally, when the number of autoregulatory interactions increases, the robustness against protein interaction mutations decreases, but against regulatory mutations increases (Figure 3C).

### Integration with biological data

The best way to validate our predictions would be to compare gene regulatory networks across different species. In combination with expression patterns, this would shed light on how evolutionary changes of network structure do or do not result in changes in network output. In the absence of sufficient amounts of such data, we obtained indirect evidence from data on biological networks. Three of the five predictions summarized in Table 1 are consistent with such data. For two of these, our reasoning was that network features which are less robust upon changes should be more conserved. First, we predicted that networks are more robust against regulatory mutations than against protein interaction mutations. This is consistent with the observation that protein-protein interactions are more conserved than regulatory interactions [33], [34], and although this is well-known, the fact that our simulations reproduce it is reassuring for our approach. Second, we predicted more robustness against changes in repressive interactions; indeed it has been reported that activators are somewhat more frequently conserved among bacterial genomes than repressors [35]. That same study also reported that mixed activators/repressors have the highest conservation, which would suggest the lowest robustness upon mutation, following the reasoning that network features which are less robust upon changes should be more conserved. Indeed we observed in part of our network ensembles, although not in all of them, that mixed regulators lead to lowest robustness, depending on the topology parameters. However, in our analysis indeed mixed regulators appeared to have lower robustness than pure repressors for all topology parameters (details are reported in Text S1, Table S2). Third, the existence of compensatory mutations is supported by various lines of evidence [27], [36], [37], [38]. For example, the evolution of alternative transcriptional circuits with identical output logic has been described [36], and a study on combinatorial gene regulation reported turnover of cis-acting sequence and the formation of new protein-protein interactions [37]. Similarly, analysis of allele-specific expression in parental and hybrid strains revealed the existence of compensating mutations in various species [38], [39]. In this last example, contrary to the first two examples, it is known that the individual mutations indeed have significant effects.

For our prediction about autoregulatory interactions, where we found an opposite dependence of robustness against protein interactions mutations or against regulatory mutations on the number of such autoregulatory interactions, we did not find data for falsification or verification. However, if our prediction is correct, it could be an important aspect of gene regulatory network evolution that robustness against different types of mutations requires a different optimal fraction of autoregulatory interactions. In general, autoregulatory interactions are known to be important for various aspects of regulatory networks, including speeding up response times [40] and providing robustness against fluctuations in concentrations [41], and hence how mutational robustness of gene regulatory networks is influenced by autoregulatory interactions is an important issue.

**Figure 4:**
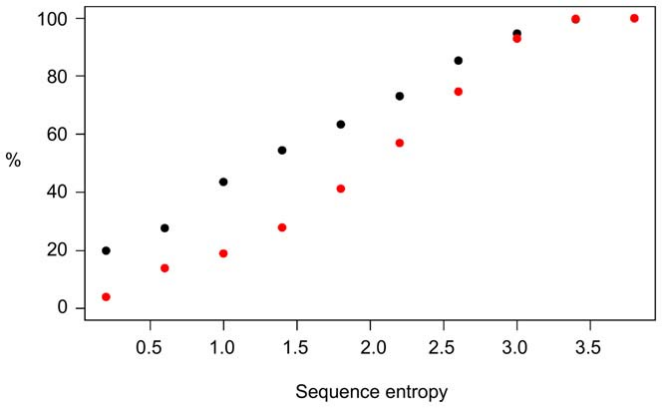
Cumulative histogram of sequence conservation of DNA contacting residues in human TFs. Sequence entropy was calculated for dimeric (black) and monomeric TFs (red). Lower values indicate more conservation.

Finally, a novel and remarkable prediction is that mutations in regulatory interactions lead to larger changes in networks with dimeric TFs compared to networks with monomers. Given the importance of TF-TF protein-protein interactions as explained above, this difference in robustness could be an important aspect of regulatory interaction network evolution. For this predicted difference in robustness, we obtained indirect validation by analyzing differences in conservation of DNA-contacting residues in monomeric and dimeric TFs. Here we followed again the reasoning that features which are connected to lower robustness should be more conserved. Hence, based on our prediction, we expect on average more conservation of dimeric DNA-contacting residues, compared to the conservation of such residues in monomeric TFs. To test this hypothesis, we analyzed the distributions of sequence entropy values for DNA-contacting residues in both types of TFs, using a set of 57 monomeric and 228 dimeric human TFs for which structural information was available (Figure 4). Indeed, these were significantly different as tested using a Kolmogorov-Smirnov test (p<10^−15^). Dimeric DNA-contacting residues display lower entropy, i.e. higher conservation, in accordance with our hypothesis. Such differences were not observed for residues in those TFs that do not contact DNA.

## Discussion

Robustness in biological networks can have various origins. First, redundancy of gene functions is important, as genes with overlapping functions will be able to compensate for each other. A second source is robustness that has its origin in interactions between genes with unrelated functions, i.e. different positions in the network, not having the same set of interactions. Such robustness is a property of the network as a whole and not solely of some of the components. Some studies indicated that such “distributed robustness” would be more important than redundancy for robustness against mutational loss in yeast [42], [43], although this has been challenged [44]. In our analysis this second type of robustness is what we look at. Our model is relatively simple; in particular, there is no explicit coupling between dimerization and DNA interaction specificity. However, compared to existing approaches using Boolean Networks or related approaches our use of ODEs means that we can study more biological relevant aspects, in particular explicit dimerization.

We focused on obtaining evidence for factors influencing robustness against mutations of network structure. The results of our analysis provide several clues towards such factors. An important follow-up would be to investigate in detail the mechanistic aspects of why certain types of networks are more robust than others. Although we will leave that question to a large extent to be addressed by future work, we briefly discuss two relevant issues. One is related to the observation that regulatory mutations in dimer networks have larger effect than in monomeric networks. This might be related to the fact that in dimeric networks, the change in expression of a target gene caused by a regulatory mutation will not only influence other genes via regulatory interactions of that target, but also via protein interactions that it is involved in. We obtained evidence for this by analyzing whether the regulatory mutation mainly had a direct effect (changing expression level of the target gene targeted by the mutation) or mainly an indirect effect (changing expression levels of other genes). In dimeric networks, the indirect effect was much larger than the direct effect, whereas for monomeric networks, the indirect effect was somewhat smaller than the direct effect. Comparing dimeric and monomeric networks, the direct effects were roughly comparable, whereas the indirect effect was much larger in the dimeric network (data not shown). Although this should be analyzed in more detail, this analysis indicates a causal mechanism for the observed differences in robustness between dimeric and monomeric networks. A second point is related to the fact that repressive interactions confer more robustness; here one could argue that the effects of a mutation in an interaction which tends to switch off expression of a target gene, will be less easily transferred to other parts of the network, compared to a mutation in an interaction that activates a target gene, because in the latter case the product that has been upregulated might have all kinds of additional effects.

In addition to regulatory interactions, protein-protein interactions are an important type of connections in gene regulatory networks [4]. Our analysis provides insight into how mutations of both types of interactions can shape network evolution. We studied compensation between protein-protein and regulatory mutations; such compensatory mutations have indeed been observed experimentally, as mentioned above. These observations so far constituted isolated examples, but our results highlight the potential general importance of this type of interplay between protein-protein interaction mutations and regulatory mutations, providing a framework for further study of such effects. In addition, based on observed differences in robustness in our simulations, we predicted differential conservation of DNA-contacting residues in monomeric vs. dimeric TFs, a prediction which we validated using protein structure and sequence data.

The integration and comparison with biological data that we perform is based to some extent on the hypothesis that network features which induce lower robustness upon mutations to network structure should be more conserved. Obviously, this hypothesis needs not always hold, because for some processes and in some circumstances evolutionary change and not robustness will be favorable and will be selected. Nevertheless, the trends we find are consistent with biological datasources. For one of those, the fact that TF-DNA interactions are less conserved than protein-protein interactions, it is important to realize that this does not necessarily mean that DNA binding domains of TFs are less conserved than protein interaction domains. In fact, the opposite has been observed in an analysis that focused on conservation of DNA binding domains (although not specifically on residues involved in DNA binding) [4]. However, because of rapid turnover of TF-binding cis-motifs [33], [34], [45], at the network level the net effect is still that regulatory interactions are less conserved than protein-protein interactions [33], [34].

Our results clearly demonstrate the importance of taking protein-protein interactions into account when studying the robustness of gene regulatory networks. Although much attention in studying gene regulatory networks and their evolution has been focused on regulatory interactions, recently data is coming available which integrates TF-TF interactions within the context of such networks [46]. In addition, powered by the ongoing revolution in sequencing technologies and their application in e.g. ChIP-seq experiments, large amounts of data on transcriptional regulatory interactions and the evolution of network connections are being generated [47], [48], [49]. Our analysis provides a framework for further study of these networks and their evolution. In particular, previous studies indicated the importance of functional constraints on network topology, such as the importance of different types of topologies to obtain either multistationarity or homeostasis [50]. Our results implicate the existence of additional evolutionary constraints.

## Methods

### Simulations of network models

Network models were simulated using ordinary differential equations (ODEs) where monomer and dimer concentrations were represented explicitly. The rate of change of the dimer concentrations consists of the association rate of monomers into dimers minus the dissociation rate of dimers into monomers, minus the dimer decay rate. Denoting by x_i_ the concentration of monomer i and by x_ij_ the concentration of a dimer consisting of proteins i and j, we have the following equation in case of a single dimer:
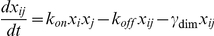
Here k_on_, k_off_ are the forward and backward dimerization rate constants and γ_dim_ is the dimer decay rate. Extensions in the more general case where multiple dimers are formed are presented in Text S1.

For the monomer dynamics, activating or repressing Hill functions were used to model gene regulation, which are combined with dimerization reactions and monomer decay. In the case of regulation by one particular dimer, for a protein involved in one dimerization reaction, we have the following equations:
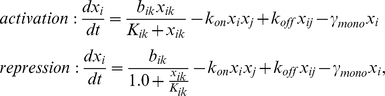
for activation and repression, respectively. Here b_ik_ and K_ik_ are Hill function parameters for the regulation of gene *i* by dimer *k* (in a more general setting one could also include a cooperativity parameter but we set this to 1). γ_mono_ is the monomer decay rate. Extensions for more general cases are shown in Text S1.

In order to compare the simulated systems with dimeric protein-protein interactions, also networks were simulated with only monomeric proteins. These monomers directly regulate transcription, again via Hill functions (equations for monomeric systems are provided in Text S1)

Parameters that occur in those equations were randomly chosen in various network realizations, but were limited to certain ranges or values (Table 2). These were based on literature values [7], [51], [52]. Parameters that describe the topology of the systems are shown in Table 2 as well. The fraction of dimers is calculated as follows: f_dim_ = n_dim_/(0.5*n_prot_^2^+0.5*n_prot_). Here n_dim_ is the number of dimers present in the network. The fraction of regulatory interactions (F_reg_) is the average number of dimers or monomers regulating a gene.

**Table 2:** Network model parameters and network topology parameters.

Parameter	Description	Value or range
**Model parameters**		
γ	Degradation rate	0.2 min^−1^ for monomer; 0.01 min^−1^ for dimer
k_off_	Dimerization off rate	0.01 min^−1^
k_on_	Dimerization on rate	10^−2^ or 1 nM^−1^ min^−1^
b	Hill function maximal expression level	Within range 40 nM min^−1^–2400 nM min^−1^
k	Hill function activation coefficient	Within range 10 nM–1000 nM
**Topology parameters**		
N_prot_	Number of proteins	6
F_dim_	Fraction of dimers	0.0, 0.3 or 0.6
F_regint_ a	Fraction of regulatory interactions	2.0 or 4.0
F_act_ b	Fraction of regulatory interactions that is activating	0.25, 0.5 or 0.75

a F_regint_ is calculated as the number of regulatory interactions per protein.

b F_act_ is calculated as the ratio of the number of activating regulatory interactions over the total number of regulatory interactions.

We investigated how the robustness of the network output depends on the topology parameters in randomly generated networks. To analyze this, two test sets were generated: 1) set1, with a limited number of networks where a large number of ODE parameter assignments could be tested, and 2) set2, with a much larger number of networks, without sampling of the ODE parameters for each network. For set1, 20 different networks were generated with random topology for each combination of parameters in Table 2, except that F_regint_ was only set equal to 2.0, giving 120 networks in total (2×3×20). The network topology was generated using the selected values of interaction densities as probabilities for each possible protein-protein interaction or regulatory interaction, ensuring however that each gene is regulated by at least one dimer or monomer. For each of these networks, 1,000 random assignments were generated for Hill parameters, k_on_ and starting concentrations. This set was used for the analysis of compensatory mutations.

Set2 was used for the other analyses; here, much larger ensembles of networks were generated, using all possible combinations of network parameters shown in Table 2. In each such ensemble, 25,000 different topologies were generated, all with the same number of proteins, same fraction of dimers and same fraction of regulatory interactions, but each with different connections. For each such network, parameters were initialized randomly within the boundaries indicated in Table 2.

Simulations were started using only monomers present; their values were set to randomly chosen values within the range of 1 nM–100 nM. The simulation time was 15,000 min. ODEs were solved using Biocham [53] with the fourth-order Runge-Kutta method, adapting the step size to a maximum error of 1E-10 and using the default initial step size of 0.01.

To investigate the stability upon network rewiring, the following simulations were performed. For each parametrized system of ODEs, one ‘wildtype’ simulation was done. Then, independently from each other, two mutations were performed changing network structure: (1) a protein-protein interaction change, where a dimer A–B is changed to dimer A–C (that did not occur yet in the protein-protein interaction network); this new dimer keeps exactly the same regulatory interactions as were previously attached to the deleted dimer; (2) a regulatory change where for one specific dimer, one of the genes that it regulates is changed to another gene (which it did not yet regulate). All parameters remain unchanged when performing those mutations; the ‘new’ interaction takes over the parameters from the ‘old’ interaction. Figure 1 provides a graphical illustration of the mutations. The way in which these mutations were constructed ensures that the number of interactions does not change, which allows a more fair comparison between wildtype and mutated system than in case this number would change. The mutated systems were simulated as well, and the resulting concentrations were compared with the wildtype concentrations.

To assess the change after mutation, a relative change was defined as follows (D_mut_):
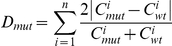
Here the summation goes over all concentrations (i.e. *n* equals the number of monomers plus the number of dimers), C_wt_ is the wildtype concentration and C_mut_ the value observed in the mutated system.

### Expression data analysis

An important methodological question is how to determine a suitable cutoff for D_mut_ such that the difference between the expression patterns is “small” or “large”, i.e. a cutoff D_small_ and a cutoff D_large_. To answer this question, we calculated values obtained for D_mut_ by analyzing a set of different gene expression datasets. These included two inter-species comparisons, using expression data for four different wine yeast strains [28] and for two *Drosophila* species [29], and two intra-species comparisons (comparing different cell types) within human [31] and within *Arabidopsis thaliana* [30]. For the latter, a focused set of factors involved in flowering and floral organ determination was used: AG, AP3, PI, AP1, SHP1, SEP3, WUS, LFY, UFO, miR172, SUP, LUG, SEU, SAP, RBE, YUC2, YUC6, YUC1, YUC4, PIN1, NPY1, BLR, CUC1, whereas for human the dataset consisted of genome-wide expression data across several tissue. For all datasets, we randomly selected 6 proteins 1,000 times and computed their D_mut_ values between two different conditions (different species or different tissues).

Note that multiplicative normalization of microarray data does not influence the calculated D_mut_. However, log transformation does, so in case of the *Drosophila* data we back-transformed the log transformed data.

### Statistical significance of observed differences in network robustness

Statistical significance of the observed differences in robustness between protein interaction vs. regulatory changes was assessed using a randomization procedure. Here, the observed values of D_mut_ for the mutated networks were randomly reassigned to ‘protein interaction’ or ‘regulatory interaction’ mutation. Next, the percentage of rewired networks with only a small change (D_mut_<D_small_) was calculated for these randomized datasets, and the difference between those percentages for protein interaction vs. regulatory interaction mutations in the randomized datasets was compared to the observed difference obtained with the input dataset. This was repeated 1,000 times. A p-value was obtained as the number of cases in which this difference was at least as large in the randomized datasets as compared to what was obtained with the input datasets. In a similar way, the significance for the difference in robustness observed for regulatory changes in monomeric vs. dimeric networks was assessed.

### Compensatory mutations

For mutations that produced a large change (D_mut_>D_large_) we tested if compensatory mutations could be found that to a large extent rescued the phenotype of the network. A second mutation was applied, and all parameter values remained unchanged. This was tested with set1 described above, applying 1,000 regulatory and 1,000 protein interaction mutations as secondary mutations to each primary high-effect mutation.

### Network properties

The network ensembles we generated were based on ‘simple’ topological parameters (number of proteins, number of interactions). More involved parameters might offer a more comprehensive description of network properties of relevance for mutational stability. One example is regulation entropy, which was calculated as described in ref. [18]. Briefly, first all regulatory paths between two nodes are obtained (for which we simply did an exhaustive search which is very fast in our small networks). Next, each path is scored as positive if the number of repressing interactions is even, and negative if this number is odd. The entropy of the regulation of one node by another node is the entropy calculated using the fractions of positive and negative paths. The regulation entropy of a given node is the average of its regulation entropies with respect to all the other nodes.

For the networks without dimerization this calculation is identical to that in ref. [18]. However, for the dimeric systems, one has to choose how to treat dimers when finding regulatory paths between two nodes. We simply used the most straightforward approach, consisting of ‘splitting’ each dimer such that for each dimeric regulatory interaction, the path finding in fact finds two branches. Regulation entropy did not show clear relationship with robustness and is not presented in the Results section.

In addition to regulation entropy, we also used the number of autoregulatory interactions as a characteristic of the network. In this case, only direct interactions are used. As with regulation entropy, for each dimer we ‘splitted’ the regulatory interaction such that if a dimer A–B regulates both A and B, there would be two autoregulatory interactions.

As a third parameter, redundancy of interactions was calculated as a proxy for gene function redundancy. Here, we calculated interaction similarity, for dimer interactions, for regulatory interactions and for both types of interactions. Interaction similarity was defined as the number of similar interactions divided by the number of different interactions plus the number of similar interactions. We analyzed both average and maximum interaction similarity. This did not result in any clear dependence of robustness on redundancy; hence, this analysis is not presented in the Results section.

### Structure analysis

To test the hypothesis that DNA-contacting residues in dimeric TFs are more conserved than those in monomeric TFs, a set of human dimeric and monomeric TFs was assembled using information from the HPRD database [54]. We compared TFs (obtained by using the GO term 0003700, transcription factor activity) that at least interact with one other TF according to the HPRD data with those TFs that do not interact with any other TF. The TFs were mapped to available protein structures, as obtained from the GTOP database [55]. DNA contacting residues were defined using a cutoff of 10 Å (using 5 Å instead did not change the results). Conservation was assessed on sequences obtained by using blast [56] vs. the NR database and keeping one randomly chosen sequence per species (with a blast E-value cutoff of 1E-25). As a measure of conservation, sequence entropy was calculated.

## Supporting Information

Text S1 **Contains information about simulation setup and details of results.**
(PDF)

Figure S1 **Percentage of networks whose output does not change upon mutation (D_mut_<D_small_).** Fraction of dimers (F_dim_) equals 0.0, 0.3 or 0.6, and fraction regulatory interactions (F_regint_) equals 2.0 or 4.0. Blue, protein interaction mutations (only for cases with F_dim_>0); red, regulatory mutations. (**A**) Fraction activating interactions 0.25. (**B**) Fraction activating interactions 0.5. (**C**) Fraction activating interactions 0.75.
(TIF)

Table S1 **Robustness against protein interaction vs. regulatory interaction mutation.**
(PDF)

Table S2 **Robustness against mutations in repressive vs. activating regulators.**
(PDF)
