# Supplementary material for: Mutational Robustness of Gene Regulatory Networks
Source: PLoS One. 2012 Jan 25;7(1):e30591. doi: 10.1371/journal.pone.0030591 (PMC3266278; doi:10.1371/journal.pone.0030591)
Supplement: Text S1 — Contains information about simulation setup and details of results. (PDF) [file pone.0030591.s001.pdf]

**Supplementary Information**

# Mutational robustness of gene regulatory networks

A.D.J. van Dijk[1,2,3]*, S. van Mourik[2] and R. C. H. J. van Ham[1, §]

[1] Applied Bioinformatics, PRI, Wageningen UR, Droevendaalsesteeg 1, 6708 PB Wageningen, The Netherlands

[2] Biometris, Plant Sciences Group, Wageningen UR, Droevendaalsesteeg 1, 6708 PB Wageningen, The Netherlands

[3] Netherlands Consortium for Systems Biology (NCSB), P.O. Box 94215, 1090 GE Amsterdam, The Netherlands

* Email: aaltjan.vandijk@wur.nl

* Telephone: +31.317.480994

[§] Current address: Keygene N.V., P.O. Box 216, 6700 AE Wageningen, The Netherlands

**Supplementary Information Text**

**Simulation setup**

In the Methods section of the main text we describe the ODEs used in case of dimeric systems, and assuming regulation of a gene by only one dimer. Here we present more general equations and discuss some specific choices that were made. First, in case of monomeric networks, and assuming only one regulator for a given gene, the following equations were used, respectively for an activating or a repressive interaction:

$$\frac{dx_i}{dt} = \frac{b_k \cdot x_k}{K_k + x_k} - \gamma \cdot x_i$$

$$\frac{dx_i}{dt} = \frac{b_k}{1.0 + \frac{x_k}{K_k}} - \gamma \cdot x_i$$

Here, $x_i$ indicates the concentration of the target gene, $x_k$ that of the regulator, $\gamma$ the decay constant and b and K indicate Hill function parameters.

Multiple regulators were combined by adding the different Hill function terms. This leads for example to the following equation for a monomeric system where a given gene is regulated by one activator and one repressor:

$$\frac{dx_i}{dt} = \frac{b_k \cdot x_k}{K_k + x_k} + \frac{b_l}{1.0 + \frac{x_l}{K_l}} - \gamma \cdot x_i$$

Similarly, for a dimeric network the equation for a gene regulated by one activator and one repressor, and involved in two different dimers, looks as follows:

$$\frac{dx_i}{dt} = \frac{b_{ik} \cdot x_{ik}}{K_{ik} + x_{ik}} + \frac{b_{il}}{1.0 + \frac{x_{il}}{K_{il}}} - k_{on}x_i x_m + k_{off}x_{im} - k_{on}x_i x_n + k_{off}x_{in} - \gamma \cdot x_i$$

Here $k_{on}$ and $k_{off}$ indicate the dimerization constants, and $x_{im}$ and $x_{in}$ the concentrations of the two dimers that gene *i* is involved in. Monomeric concentrations are indicated by $x_i$, $x_m$ and $x_n$.

An important simplification in our model is that we do only explicitly represent proteins, and not RNA. Although explicitly representing RNA would add to the biological realism of our model, our choice simplifies the computation and reduces the computational time needed. In addition, compared to existing approaches to study network robustness using Boolean Networks (which also do not explicitly represent RNA), our approach is more realistic biologically, in particular because we explicitly represent protein-protein interactions.

To implement the mutations of protein-protein and of regulatory interactions, we used a python script that takes as input a file containing the description of the equations in biocham format. Randomly, one protein was chosen, and depending on the type of mutation (protein-protein or regulatory) the relevant changes in the equations were introduced.

**Results are not sensitive to exact value of cutoff for $D_{mut}$**

Results reported in main text were obtained with threshold values for $D_{mut}$ of $D_{small}$=0.2 and $D_{large}$=1.0. Here we assess whether our results are sensitive to the exact values of those cutoffs.

Observation 1 (protein interaction mutations are more likely to have a large effect) and observation 4 (networks without dimers are more robust against changes in regulatory interactions) were based on counting percentage of networks with $D_{mut}$ larger than or smaller than the threshold, and hence might potentially be influenced by the exact value of the threshold. As shown in Table S1 this is however not the case.

Observation 2 (repressive interactions are associated with a higher robustness against mutations, compared to activating interactions) and observation 5 (influence of autoregulatory interactions) were based on average values of $D_{mut}$ and hence are not influenced by the value of the threshold. Finally, for observation 3 (compensatory mutations) we discuss in the main text that there is some influence of the threshold value but that the main trend that we observed does not change when the threshold is changed.

**Mutations of repressive vs. activating interactors**

As explained in the main text, repressive interactions are associated with a higher robustness against protein-protein interaction mutations as well as regulatory interaction mutations, compared to activating interactions. This was assessed by counting the number of repressive vs. activating interactions that the regulator affected by the mutation was involved in, and assigning the regulatory to one of the categories ‘purely repressive’, ‘purely activating’, or ‘mixed’. Table S2 shows this analysis for the for each of the different values of $F_{dim}$ and $F_{regint}$ separately.

**Influence of fraction activating interactions**

Results shown in Main Text Figure 3 are obtained with fraction activating interactions 0.5 but are qualitatively similar for fraction activating interactions 0.25 or 0.75, as shown in Figure S1.
