# Supplementary figures and images for: Mutational Robustness of Gene Regulatory Networks
Source: PLoS One. 2012 Jan 25;7(1):e30591. doi: 10.1371/journal.pone.0030591 (PMC3266278; doi:10.1371/journal.pone.0030591)

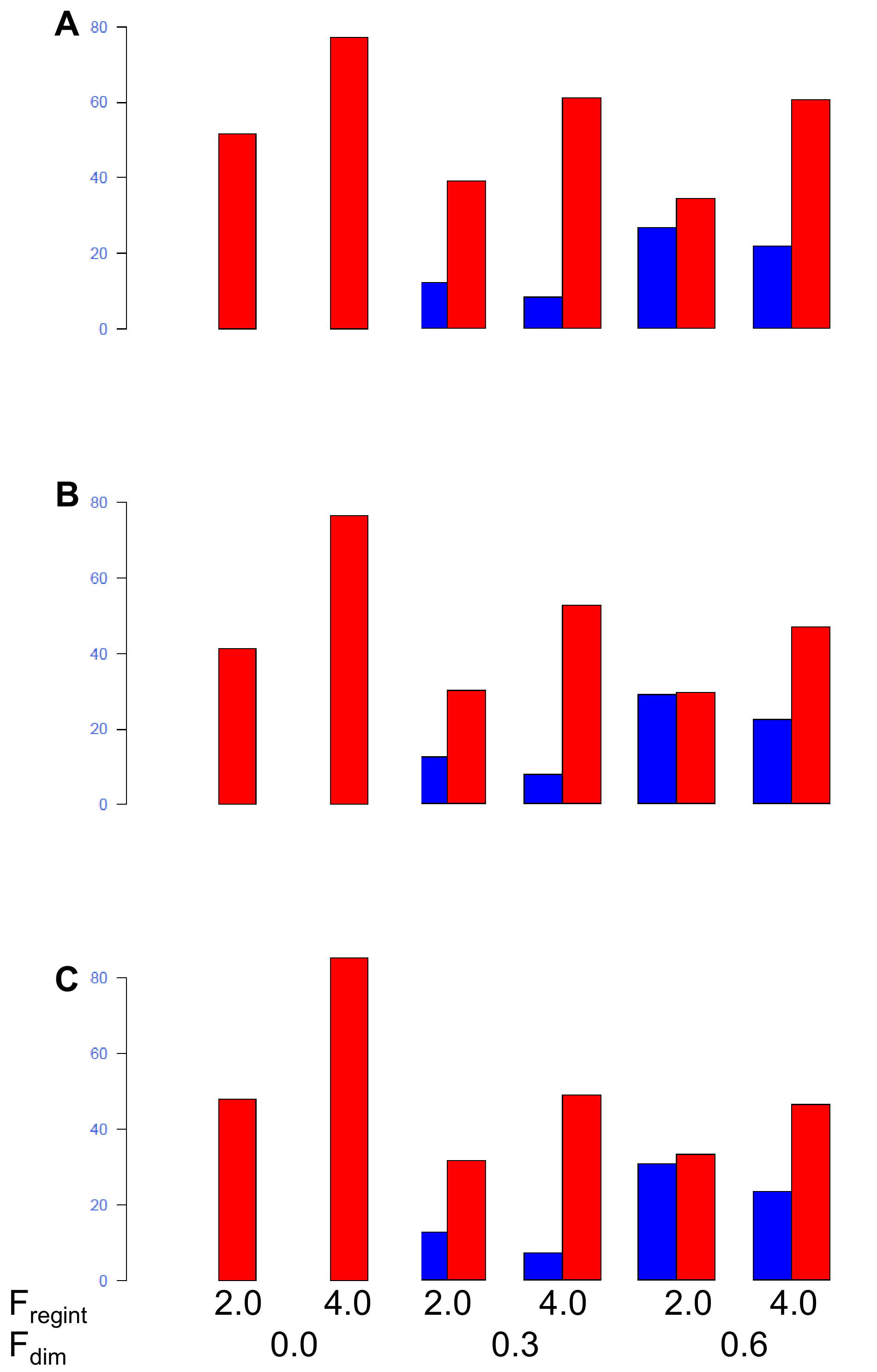

Supplement: Figure S1 — Percentage of networks whose output does not change upon mutation (Dmut<Dsmall). Fraction of dimers (Fdim) equals 0.0, 0.3 or 0.6, and fraction regulatory interactions (Fregint) equals 2.0 or 4.0. Blue, protein interaction mutations (only for cases with Fdim>0); red, regulatory mutations. (A) Fraction activating interactions 0.25. (B) Fraction activating interactions 0.5. (C) Fraction activating interactions 0.75. (TIF) [file pone.0030591.s002.tif]
