# Supplementary material for: Mutational Robustness of Gene Regulatory Networks
Source: PLoS One. 2012 Jan 25;7(1):e30591. doi: 10.1371/journal.pone.0030591 (PMC3266278; doi:10.1371/journal.pone.0030591)
Supplement: Table S1 — Robustness against protein interaction vs. regulatory interaction mutation. (PDF) [file pone.0030591.s003.pdf]

Supplementary Information

Mutational robustness of gene regulatory networks

A.D.J. van Dijk[1,2,3]*, S. van Mourik[2] and R. C. H. J. van Ham[1, §]

[1] Applied Bioinformatics, PRI, Wageningen UR, Droevendaalsesteeg 1, 6708 PB Wageningen, The Netherlands

[2] Biometris, Plant Sciences Group, Wageningen UR, Droevendaalsesteeg 1, 6708 PB Wageningen, The Netherlands

[3] Netherlands Consortium for Systems Biology (NCSB), P.O. Box 94215, 1090 GE Amsterdam, The Netherlands

* Email: aaltjan.vandijk@wur.nl

* Telephone: +31.317.480994

[§] Current address: Keygene N.V., P.O. Box 216, 6700 AE Wageningen, The Netherlands

Table S1. Robustness against protein interaction vs. regulatory interaction mutation.

F_{dim}	F_{regint}	Protein interaction[a]	Regulatory interaction[a]
**D_{small} =0.2**			
0.0	2.0	N/A	41.20
0.0	4.0	N/A	76.40
0.3	2.0	12.33	29.96
0.3	4.0	7.84	52.54
0.6	2.0	28.87	29.39
0.6	4.0	22.47	46.78
**D_{small} =0.1**			
0.0	2.0	N/A	25.20
0.0	4.0	N/A	58.00
0.3	2.0	5.98	23.86
0.3	4.0	2.99	45.61
0.6	2.0	14.13	24.31
0.6	4.0	10.46	38.02
**D_{small} =0.5**			
0.0	2.0	N/A	78.00
0.0	4.0	N/A	97.20
0.3	2.0	32.11	46.63
0.3	4.0	27.15	70.25
0.6	2.0	46.79	45.50
0.6	4.0	44.85	65.23

[a]Mutations were introduced on either protein interaction or regulatory interactions, and the percentage of networks for which network output was robust ($D_{mut}<D_{small}$) is reported.
