# Supplementary material for: Mutational Robustness of Gene Regulatory Networks
Source: PLoS One. 2012 Jan 25;7(1):e30591. doi: 10.1371/journal.pone.0030591 (PMC3266278; doi:10.1371/journal.pone.0030591)
Supplement: Table S2 — Robustness against mutations in repressive vs. activating regulators. (PDF) [file pone.0030591.s004.pdf]

Supplementary Information

Mutational robustness of gene regulatory networks

A.D.J. van Dijk[1,2,3]*, S. van Mourik[2] and R. C. H. J. van Ham[1, §]

[1] Applied Bioinformatics, PRI, Wageningen UR, Droevendaalsesteeg 1, 6708 PB Wageningen, The Netherlands

[2] Biometris, Plant Sciences Group, Wageningen UR, Droevendaalsesteeg 1, 6708 PB Wageningen, The Netherlands

[3] Netherlands Consortium for Systems Biology (NCSB), P.O. Box 94215, 1090 GE Amsterdam, The Netherlands

* Email: aaltjan.vandijk@wur.nl

* Telephone: +31.317.480994

[§] Current address: Keygene N.V., P.O. Box 216, 6700 AE Wageningen, The Netherlands

Table S2. Robustness against mutations in repressive vs. activating regulators

F_{dim}	F_{regint}	Average D_{mut} (standard deviation)		
		**Pure repressive regulator**	**Pure activating regulator**	**Mixed regulator**
0.3	2.0	0.41 (0.45)	0.75 (0.50)	0.63 (0.52)
0.3	4.0	0.13 (0.29)	0.58 (0.46)	0.36 (0.43)
0.6	2.0	0.54 (0.49)	0.64 (0.52)	0.64 (0.51)
0.6	4.0	0.23 (0.37)	0.53 (0.44)	0.40 (0.44)
